# 

**Published:** 1882-08

**Authors:** 


					﻿Whole Number,
/ CCCCLI.
August, 1882.
Number 2,
Vol. XLV.
THE
CHICAGO
MEDI CAL
I ournal and Examiner
EDITORS:
N. S. DAVIS, M.D., LL.D. JAS. NEVINS HYDE, A.M., M.D.
DANIEL R. BROWER M.D.
CHICAGO:
PUBLISHED BY THE CHICAGO MEDICAL PRESS ASSOCIATION,
188 Clark Street.
JfRead the Notice of this Journal among Advertisements*
				

